# Comparing Contributions of Passive and Active Tick Collection Methods to Determine Establishment of Ticks of Public Health Concern Within Illinois

**DOI:** 10.1093/jme/tjab031

**Published:** 2021-04-15

**Authors:** Lee Ann Lyons, Mary E Brand, Peg Gronemeyer, Nohra Mateus-Pinilla, Marilyn O’Hara Ruiz, Chris M Stone, Holly C Tuten, Rebecca L Smith

**Affiliations:** 1 Department of Pathobiology, University of Illinois, 2001 S. Lincoln Avenue, Urbana, IL 61802, USA; 2 Illinois Natural History Survey-Prairie Research Institute, University of Illinois, 1816 S. Oak Street, Champaign, IL 61820, USA; 3 U.S. Department of Agriculture, Natural Resource Conservation Service, 1211 Old 6 Road, Malcom, IA 50157, USA

**Keywords:** tick, distribution, establishment, surveillance, Illinois

## Abstract

In Illinois, between 1990 and 2017, tick-borne diseases in humans increased 10-fold, yet we have insufficient information on when and where people are exposed to vector ticks (Ixodida: Ixodidae). The aims of our research were to compare contributions of passive and active tick collection methods in determining establishment of ticks of public health concern and obtain information on tick distributions within Illinois. We used three surveillance strategies within the Illinois Tick Inventory Collaboration Network to gather information about the ticks of public health concern: 1) passive collection (voluntary submission by the public); 2) systematic collection (biweekly active surveillance); and 3) special collections (active collections in locations of special interest). Of collected adult and nymphal ticks, 436 were from passive collections, 142 from systematic collections, and 1,270 from special collections. Tick species distribution status changed in 36 counties. Our data provide noteworthy updates to distribution maps for use by public health agencies to develop prevention and control strategies. Additionally, the program built a network of collaborations and partnerships to support future tick surveillance efforts within Illinois and highlighted how the combination of the three surveillance strategies can be used to determine geographic spread of ticks, pinpoint locations in need of more surveillance, and help with long-term efforts that support phenology studies.

Tick-borne diseases (TBDs) make up the majority of locally acquired vector-borne disease cases reported to the Centers for Disease Control and Prevention (CDC) each year in the continental United States (Adams et al. 2016, Eisen et al. 2017b). The predominant causative agents for these TBDs are bacterial pathogens from the genera *Borrelia* and *Rickettsia*. Lyme disease (*Borrelia burgdorferi*) is the most common TBD reported in people, while the deadliest in the United States is Rocky Mountain spotted fever (*Rickettsia rickettsii*) (Eisen et al. 2017b, Rosenberg et al. 2018). Anaplasmosis caused by the bacterium *Anaplasma phagocytophilum* and ehrlichiosis cause by the bacteria *Ehrlichia chaffeensis*, *Ehrlichia ewingii*, or *Ehrlichia muris eauclairensis* have also increased in recent years, especially in the Upper Midwest (Johnson et al. 2015). Within Illinois, the number of reported cases of illness from the four most common TBDs (Lyme disease, Rocky Mountain spotted fever, ehrlichiosis, and anaplasmosis) jointly increased almost 10-fold between 1990 and 2017 (Illinois Department of Public Health [IDPH] 2017a, b, 2018; CDC 2018). Additionally, Heartland virus was recently documented in humans and ticks in the state (Tuten et al. 2020).

Increased incidence of TBDs in Illinois is related to expanding geographical ranges of hard-bodied (Ixodida: Ixodidae) vector ticks (Eisen and Eisen 2018, Gilliam et al. 2020, Lehane et al. 2020). Currently, Illinois is on the frontlines of geographic expansion for multiple tick species (Rydzewski et al. 2011, Springer et al. 2014, Lockwood et al. 2018, Phillips et al. 2020, Tuten et al. 2020). The species of most serious concern are the American dog tick [*Dermacentor variabilis* (Say) (Ixodida: Ixodidae)], which is thought to be established throughout the state, the lone star tick [*Amblyomma americanum* (L.) (Ixodida: Ixodidae)], the Gulf coast tick [*Amblyomma maculatum* (Koch) (Ixodida: Ixodidae)], a recent invader, and the blacklegged tick [*Ixodes scapularis* (Say) (Ixodida: Ixodidae)] (Phillips et al. 2020). The preferred habitat, feeding preferences, and the ability to carry and transmit infectious agents vary among tick species and influences how the ticks and pathogens expand into new areas (Eisen et al. 2017a).

Recent research conducted in Illinois used historical data from 1905 to 2017 to determine the geographic distribution of *D. variabilis*, *A. americanum*, and *I. scapularis* (Gilliam et al. 2020). The county status was categorized using the same county-scale criteria as Dennis et al., in which at least six ticks of any given life stage or at least two life stages must be identified within 12 mo to declare a county ‘established’ for a particular species (Dennis et al. 1998). Even after collating the historical tick data available, many counties throughout Illinois were still in the ‘no data’ or ‘reported’ categories for the three species of interest. There could be multiple reasons for this including: the tick species are not (yet) established in those counties; the ticks became established after researchers had discontinued surveillance; enough ticks have been found in those counties to say they are established but it was not reported; or no one has looked for the ticks in those counties.

Two different surveillance strategies can be used to estimate tick distributions: passive and active. Passive tick surveillance consists of people submitting ticks to researchers or government agencies found on themselves, their pets, or their property when the tick encounter was incidental (Koffi et al. 2012). While this method sometimes lacks geographic and temporal precision, it can be used to determine tick distribution status and has been shown to be an indicator of tick abundance and human exposure in the environment (CDC 2019, Ripoche et al. 2018). Active surveillance is systematic field collection using dragging or flagging sampling, walking surveys, removal of ticks from hosts, or CO_2_ trapping (CDC 2019). Drag sampling is considered the most quantitative method for collecting host-seeking *I. scapularis* nymphs but is also effective for collecting other vector species and life stages (Falco and Fish 1992, Rynkiewicz and Clay 2014).

While research and tick collection efforts reach back to the early 1900s in Illinois, most efforts have been focused on determining the distribution of *I. scapularis* in the northern half of the state (Rydzewski et al. 2011, Hamer et al. 2012, Rydzewski et al. 2012, Schneider et al. 2015, Eisen et al. 2016). Additionally, even though ticks from Illinois have been submitted to multiple passive surveillance programs throughout the country, Illinois previously lacked its own statewide passive tick surveillance program. Tick surveillance functions to provide information on when and where people are at risk for exposure to ticks and tick-borne pathogens, explain and predict trends in risk for TBDs, and plan future public health efforts related to TBDs (Eisen and Paddock 2021). The aims of our research were to compare contributions of passive and active tick collection methods in determining establishment of ticks of public health concern and obtain information on tick species distributions within the state. Three surveillance strategies were used within the Illinois Tick Inventory Collaboration Network (I-TICK) to gather information about the ticks of public health concern: 1) passive collections, 2) systematic active collections, and 3) special active collections.

## Materials and Methods

Tick species establishment within a county is used by the CDC for determining distribution. All three of the surveillance strategies used in this study can be used to determine establishment (CDC 2019, 2020).

### Passive Collections

The I-TICK passive surveillance program, in 2018, consisted of a network of hubs and participants who sent ticks found on humans or pets located within Illinois back to the University of Illinois at Urbana-Champaign (UIUC) for species identification (presented here) and pathogen testing (Tuten et al., in preparation). Hubs consisted of centralized locations within a county, such as local health departments, forest preserve offices, or University of Illinois Extension offices, where tick collection kits could be picked up or dropped off by individual volunteer participants. Ideally, we wanted to establish a hub in every county of the state, but the program was voluntary. The organizations we were targeting as hubs were mostly underfunded government organizations or organizations that rely on volunteers. This often makes it difficult to commit even small amounts of time and resources to projects that are not required. Additionally, while we were able to accurately budget for the cost of making the tick collection kits, we were conservative in our budgeting for shipping costs due to the unknown nature of how many completed kits would be returned. Based on these two reasons, we set a goal of 30 hubs for the first year. Hubs agreed to distribute kits to willing participants and mail completed kits back to UIUC. The cost of the kits and mailing were prepaid by the I-TICK program. The participants included people whose work or leisure took them outdoors where they were likely to encounter ticks, with or without a companion animal; there was no screening or exclusion process for participants.

Each tick collection kit sent to hubs and/or participants included instructions on what information to record and submit, a data sheet for 5 d (not necessarily consecutive) of collections, five labeled vials filled with 90% ethanol, disposable tweezers, and instructions on how to remove attached ticks properly. Participants recorded the date of outdoor activity, approximate location(s) visited, hours outdoors, number of ticks found, the vial label identification associated with ticks from a specific date, and whether repellent was used. Information on the number of collection days, number of hours outdoors, and the calculated collection effort (hours outdoors divided by number of adult and nymphal ticks) per county is summarized in Supp Table 1 (online only) but is not analyzed in this paper. The kits were returned to a hub which then mailed them back to UIUC. Only ticks from vials associated with locations within a single county were used to determine county status. Additionally, ticks with engorgement status suggestive of attachment lasting longer than 2 d were excluded from determining county status. Because travel history was not asked for, we developed this method to exclude ticks that could have possibly come from outside the county listed by the participant on the day of tick collection. Engorgement status of 2 d or more suggests the tick was on the participant for longer than a day.

### Systematic Collections

**Fig. 1.**
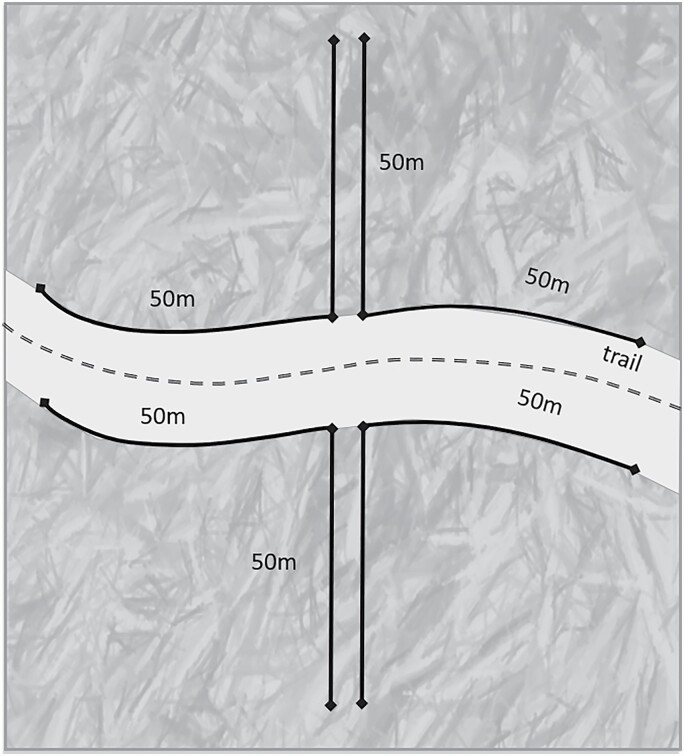
Visual representation of the systematic active surveillance tick dragging methods performed at each transect in 2018.

Systematic active tick collections were conducted in three counties (Macon, Piatt, and Champaign) of central Illinois between May and November 2018. The counties were selected based on proximity to UIUC campus, prior studies, and suitability for a related research project (Rydzewski et al. 2011, Schneider et al. 2015). Two natural areas within each county were chosen based on availability of three habitat types (i.e., maintained forest: natural area employees removed non-native species and control of competing vegetation; unmaintained forest: removal of non-native species and control of competing vegetation has not occurred within past 3 yr; and grassland) for a total of 18 sampling sites (2 natural areas × 3 habitats × 3 counties = 18). All the natural areas were under the jurisdiction of their respective county’s forest preserve district, and research permits were obtained for each. Questing ticks were collected every 2 wk (weather permitting) via dragging techniques (Falco and Fish 1992, Diuk-Wasser et al. 2006). A 1-m^2^ modified ‘finger’ drag made of white bull denim attached to a wooden dowel was used for collection (Bouseman et al. 1990). A distance collection method was employed. Tick drags were pulled along vegetation for 100 m on each side of a trail and 100 m along each side of a transect perpendicular to the trail for a total of 400 m^2^ at each site (Fig. 1). Tick drags were checked every 10–15 m, and any ticks found were placed in vials filled with 90% ethanol.

### Special Collections

We defined special collections as targeted active tick collections in locations where particular TBDs are of concern or where tick surveillance was requested by local health departments. In June 2018 (11th–15th and 29th) special collections across 42 counties in Southern Illinois were conducted. Southern Illinois was chosen due to a lack of data on tick occurrence and higher incidence of Spotted Fever Group Rickettsioses in that region compared to other parts of the state (IDPH 2017a,b, 2018; CDC 2018). We selected one natural area per county based on GIS mapping of landcover and the ability to obtain permits for tick dragging during June 2018. These natural areas included both grassland and forested habitats (whenever possible) and were a mixture of federally managed, state-managed, county/town-managed, or privately owned properties. Weather permitting, timed dragging was done along trails in both habitats. A total of 15–30 min was spent at each natural area depending on the number of questing ticks collected; drag time was extended to 30 min if fewer than 20 adult or nymphal ticks had been found. Given that only one site in one natural area was surveyed per county, the extended drag time was deemed necessary to provide stronger evidence of absence. Tick drags were checked after every minute, and any ticks found were placed in vials filled with 90% ethanol.

Additionally, three special collections, separate from the Southern Illinois collections, were done at the requests of local county health departments. These collections were done on May 24th, June 6th, and August 8th. One to two natural areas within the county were chosen by the health department personnel as areas of interest. A total of 30 min of timed dragging along trails and/or woodland boundaries was performed in each area. Drags were checked after every minute, and any adult or nymphal ticks found were placed in vials filled with 90% ethanol. All tick collection methods were approved under the biosafety protocols at the UIUC.

### Data Summarization

Adult and nymphal ticks from all three methods were collected, quantified, and identified; while larval ticks were also collected and subsamples identified, quantities were estimated only. Identifications for ticks from passive and active collections were performed at the Illinois Natural History Survey-Medical Entomology Laboratory and at UIUC Department of Pathobiology. Standard morphological identification keys were used to identify ticks to species (Mclntosh 1932, Clifford et al. 1961, Cooney and Hays 1972, Keirans and Litwak 1989, Bouseman et al. 1990, Levin et al. 1997, Keirans and Durden 1998, Dubie et al. 2017). To determine changes in vector status, ticks from all three methods were aggregated at the county level. Per Dennis et al., we determined that a tick species was ‘established’ in a county if six or more ticks of a single life stage or more than one life stage were collected (Dennis et al. 1998). A species was ‘reported’ if one to five ticks of a single life stage were collected. Tick seasonality was reported monthly for the passive surveillance and the active systematic surveillance. The active special surveillance only occurred at one time point per collection location and was not used to report tick seasonality.

ArcMap 10.7.1 was used to map active and passive collection locations and changes in distribution status (ESRI 2019). Shading was used to differentiate the previous historic status from the new status in the maps.

## Results

**Fig. 2.**
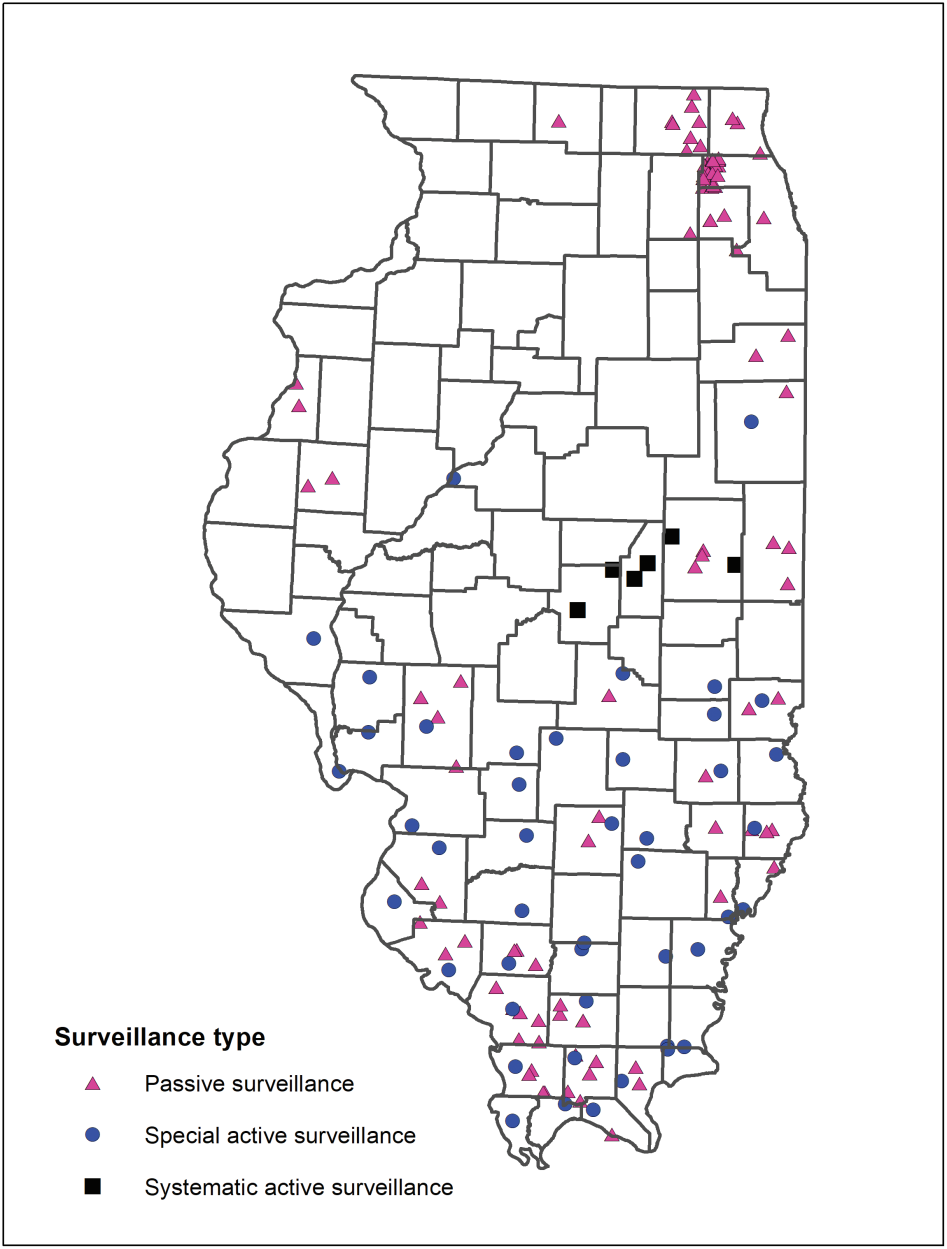
I-TICK 2018 tick collection locations.

**Fig. 3.**
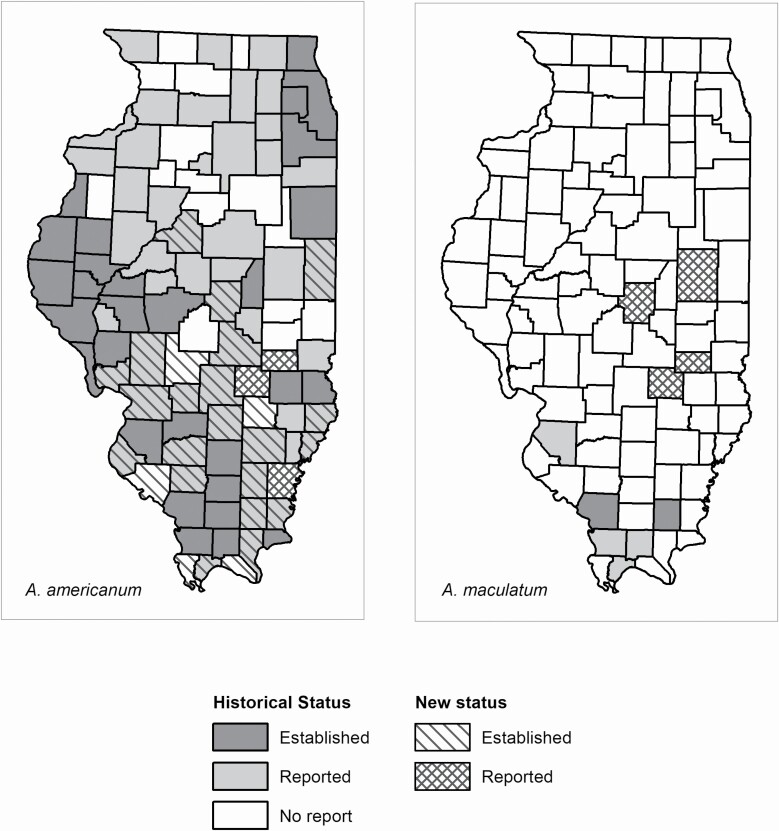
County distribution status changes for *A. americanum* and *A. maculatum* due to 2018 year of I-TICK. *Historic status data for *A. maculatum* comes from Bishopp and Trembley (1945), Gilliam et al. (2020), and Phillips et al. (2020).

**Fig. 4.**
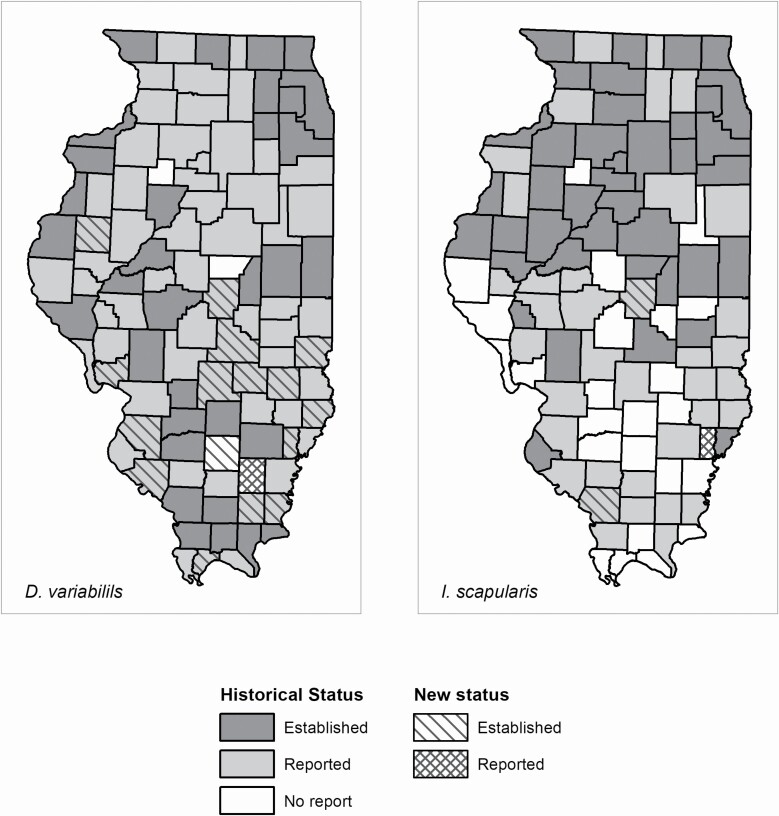
County distribution status changes for *D. variabilis* and *I. scapularis* due to 2018 year of I-TICK.

At least one of the main vector tick species was collected in 56/102 (54.9%) Illinois counties in 2018 (Fig. 2). Ticks were collected in 29/42 (69.0%) counties covered by the passive collections, 3/3 (100%) counties covered by the systematic collections, and 43/45 (95.6%) counties covered by the special collections. A total of 2,043 adult and nymphal vector ticks were collected from all three methods. Passive collections accounted for 631 of these but, after exclusion based on engorgement status, only 436 were used to determine changes to county distribution status. There were 142 adult and nymphal ticks from systematic active collections and 1,270 from special active collections. Four vector species of ticks were identified (*n* = 1,848, after passive surveillance exclusions): *A. americanum* (1,124) (Table 1); *A. maculatum* (10) (Table 2), *D. variabilis* (651) (Table 3); and *I. scapularis* (63) (Table 4). Approximately 330 larval vector ticks from three counties were collected among all three collection methods (~329 *A. americanum* and one *I. scapularis*). There were 36 counties for which tick species status (established or reported) changed for at least one of the four vector species (Figs. 3 and 4). *Dermacentor variabilis* status changed in 17 counties, *A. americanum* status changed in 29 counties, and *I. scapularis* status changed in three counties (Figs. 3 and 4). First reports of *A. maculatum* occurred in four counties (Fig. 3).

**Table 1.** Total tick numbers by county for *A. americanum* adults and nymphs from each surveillance strategy

	Passive			Special active			Systematic active			
County	Adult	Nymph	Total	Adult	Nymph	Total	Adult	Nymph	Total	Species total
Alexander	NA	NA	NA	3	19	22	NA	NA	NA	22
Bond	NA	NA	NA	0	6	6	NA	NA	NA	6
Calhoun	NA	NA	NA	3	5	8	NA	NA	NA	8
Champaign	0	0	0	NA	NA	NA	0	0	0	0
Clark	0	0	0	0	0	0	NA	NA	NA	0
Clay	NA	NA	NA	2	1	3	NA	NA	NA	3
Clinton	NA	NA	NA	4	13	17	NA	NA	NA	17
Cook	0	0	0	NA	NA	NA	NA	NA	NA	0
Crawford	NA	NA	NA	0	0	0	NA	NA	NA	0
Cumberland	NA	NA	NA	0	1	1	NA	NA	NA	1
DuPage	0	0	0	NA	NA	NA	NA	NA	NA	0
Edwards	0	0	0	0	0	0	NA	NA	NA	0
Effingham	NA	NA	NA	0	2	2	NA	NA	NA	2
Fayette	NA	NA	NA	2	4	6	NA	NA	NA	6
Franklin	NA	NA	NA	4	20	24	NA	NA	NA	24
Gallatin	NA	NA	NA	11	53	64	NA	NA	NA	64
Hamilton	NA	NA	NA	8	11	19	NA	NA	NA	19
Hardin	NA	NA	NA	19	54	73	NA	NA	NA	73
Henderson	5	0	5	NA	NA	NA	NA	NA	NA	5
Iroquois	NA	NA	NA	0	0	0	NA	NA	NA	0
Jackson	24	32	56	20	31	51	NA	NA	NA	107
Jasper	0	1	1	0	3	3	NA	NA	NA	4
Jefferson	NA	NA	NA	5	26	31	NA	NA	NA	31
Jersey	NA	NA	NA	0	10	10	NA	NA	NA	10
Johnson	2	3	5	5	17	22	NA	NA	NA	27
Kankakee	0	0	0	NA	NA	NA	NA	NA	NA	0
Lake	0	0	0	NA	NA	NA	NA	NA	NA	0
Lawrence	11	17	28	9	12	21	NA	NA	NA	49
Macon	NA	NA	NA	NA	NA	NA	1	1	2	2
Macoupin	3	0	3	0	2	2	NA	NA	NA	5
Madison	NA	NA	NA	2	2	4	NA	NA	NA	4
Marion	7	4	11	0	10	10	NA	NA	NA	21
Massac	1	1	2	0	2	2	NA	NA	NA	4
McDonough	7	1	8	NA	NA	NA	NA	NA	NA	8
McHenry	0	0	0	NA	NA	NA	NA	NA	NA	0
Monroe	NA	NA	NA	1	2	3	NA	NA	NA	3
Montgomery	NA	NA	NA	2	16	18	NA	NA	NA	18
Perry	1	3	4	3	24	27	NA	NA	NA	31
Piatt	NA	NA	NA	NA	NA	NA	0	0	0	0
Pike	NA	NA	NA	0	1	1	NA	NA	NA	1
Pope	3	1	4	15	97	112	NA	NA	NA	116
Pulaski	NA	NA	NA	19	68	87	NA	NA	NA	87
Randolph	0	2	2	1	10	11	NA	NA	NA	13
Saline	NA	NA	NA	4	39	43	NA	NA	NA	43
Shelby	0	0	0	0	7	7	NA	NA	NA	7
St. Clair	0	0	0	0	1	1	NA	NA	NA	1
Tazewell	NA	NA	NA	5	57	62	NA	NA	NA	62
Union	0	1	1	6	19	25	NA	NA	NA	26
Vermilion	2	2	4	NA	NA	NA	NA	NA	NA	4
Wabash	2	1	3	1	3	4	NA	NA	NA	7
Washington	NA	NA	NA	2	16	18	NA	NA	NA	18
Wayne	NA	NA	NA	17	42	59	NA	NA	NA	59
White	NA	NA	NA	1	0	1	NA	NA	NA	1
Williamson	10	3	13	30	62	92	NA	NA	NA	105
Winnebago	0	0	0	NA	NA	NA	NA	NA	NA	0
Species Total	78	72	150	204	768	972	1	1	2	1124

NA = counties were not sampled using surveillance strategy.

**Table 2.** Total tick numbers by county for *A. maculatum* adults from each surveillance strategy

	Passive	Special	Systematic	
County	Adult	Adult	Adult	Species total
Alexander	NA	0	NA	0
Bond	NA	0	NA	0
Calhoun	NA	0	NA	0
Champaign	1	NA	0	1
Clark	0	0	NA	0
Clay	NA	0	NA	0
Clinton	NA	0	NA	0
Cook	0	NA	NA	0
Crawford	NA	0	NA	0
Cumberland	NA	1	NA	1
DuPage	0	NA	NA	0
Edwards	0	0	NA	0
Effingham	NA	1	NA	1
Fayette	NA	0	NA	0
Franklin	NA	0	NA	0
Gallatin	NA	0	NA	0
Hamilton	NA	0	NA	0
Hardin	NA	0	NA	0
Henderson	0	NA	NA	0
Iroquois	NA	0	NA	0
Jackson	0	0	NA	0
Jasper	0	0	NA	0
Jefferson	NA	0	NA	0
Jersey	NA	0	NA	0
Johnson	0	0	NA	0
Kankakee	0	NA	NA	0
Lake	0	NA	NA	0
Lawrence	0	0	NA	0
Macon	NA	NA	4	4
Macoupin	0	0	NA	0
Madison	NA	0	NA	0
Marion	0	0	NA	0
Massac	0	0	NA	0
McDonough	0	NA	NA	0
McHenry	0	NA	NA	0
Monroe	NA	0	NA	0
Montgomery	NA	0	NA	0
Perry	0	0	NA	0
Piatt	NA	NA	0	0
Pike	NA	0	NA	0
Pope	0	0	NA	0
Pulaski	NA	0	NA	0
Randolph	0	0	NA	0
Saline	NA	0	NA	0
Shelby	0	0	NA	0
St. Clair	3	0	NA	3
Tazewell	NA	0	NA	0
Union	0	0	NA	0
Vermilion	0	NA	NA	0
Wabash	0	0	NA	0
Washington	NA	0	NA	0
Wayne	NA	0	NA	0
White	NA	0	NA	0
Williamson	0	0	NA	0
Winnebago	0	NA	NA	0
Species Total	4	2	4	10

NA = counties were not sampled using surveillance strategy.

**Table 3.** Total tick numbers by county for *D. variabilis* adults and nymphs from each surveillance strategy

	Passive surveillance			Special active			Systematic active			
County	Adult	Nymph	Total	Adult	Nymph	Total	Adult	Nymph	Total	Species total
Alexander	NA	NA	NA	3	0	3	NA	NA	NA	3
Bond	NA	NA	NA	8	0	8	NA	NA	NA	8
Calhoun	NA	NA	NA	3	0	3	NA	NA	NA	3
Champaign	15	0	15	NA	NA	NA	1	0	1	16
Clark	7	0	7	1	0	1	NA	NA	NA	8
Clay	NA	NA	NA	4	0	4	NA	NA	NA	4
Clinton	NA	NA	NA	4	0	4	NA	NA	NA	4
Cook	60	0	60	NA	NA	NA	NA	NA	NA	60
Crawford	NA	NA	NA	2	0	2	NA	NA	NA	2
Cumberland	NA	NA	NA	4	0	4	NA	NA	NA	4
DuPage	4	0	4	NA	NA	NA	NA	NA	NA	4
Edwards	11	0	11	0	1	1	NA	NA	NA	12
Effingham	NA	NA	NA	23	0	23	NA	NA	NA	23
Fayette	NA	NA	NA	17	0	17	NA	NA	NA	17
Franklin	NA	NA	NA	2	0	2	NA	NA	NA	2
Gallatin	NA	NA	NA	25	0	25	NA	NA	NA	25
Hamilton	NA	NA	NA	1	0	1	NA	NA	NA	1
Hardin	NA	NA	NA	52	0	52	NA	NA	NA	52
Henderson	0	0	0	NA	NA	NA	NA	NA	NA	0
Iroquois	NA	NA	NA	0	0	0	NA	NA	NA	0
Jackson	27	0	27	0	0	0	NA	NA	NA	27
Jasper	11	0	11	4	0	4	NA	NA	NA	15
Jefferson	NA	NA	NA	11	0	11	NA	NA	NA	11
Jersey	NA	NA	NA	8	0	8	NA	NA	NA	8
Johnson	2	0	2	1	0	1	NA	NA	NA	3
Kankakee	2	0	2	NA	NA	NA	NA	NA	NA	2
Lake	4	0	4	NA	NA	NA	NA	NA	NA	4
Lawrence	3	0	3	4	0	4	NA	NA	NA	7
Macon	NA	NA	NA	NA	NA	NA	88	0	88	88
Macoupin	11	0	11	10	1	11	NA	NA	NA	22
Madison	NA	NA	NA	2	0	2	NA	NA	NA	2
Marion	14	0	14	0	0	0	NA	NA	NA	14
Massac	0	0	0	4	0	4	NA	NA	NA	4
McDonough	15	0	15	NA	NA	NA	NA	NA	NA	15
McHenry	14	0	14	NA	NA	NA	NA	NA	NA	14
Monroe	NA	NA	NA	1	0	1	NA	NA	NA	1
Montgomery	NA	NA	NA	0	0	0	NA	NA	NA	0
Perry	5	0	5	0	0	0	NA	NA	NA	5
Piatt	NA	NA	NA	NA	NA	NA	15	0	15	15
Pike	NA	NA	NA	1	0	1	NA	NA	NA	1
Pope	1	0	1	23	1	24	NA	NA	NA	25
Pulaski	NA	NA	NA	6	0	6	NA	NA	NA	6
Randolph	6	0	6	1	0	1	NA	NA	NA	7
Saline	NA	NA	NA	6	0	6	NA	NA	NA	6
Shelby	2	0	2	20	0	20	NA	NA	NA	22
St. Clair	9	0	9	1	0	1	NA	NA	NA	10
Tazewell	NA	NA	NA	2	0	2	NA	NA	NA	2
Union	3	0	3	3	0	3	NA	NA	NA	6
Vermilion	29	0	29	NA	NA	NA	NA	NA	NA	29
Wabash	0	0	0	3	0	3	NA	NA	NA	3
Washington	NA	NA	NA	3	1	4	NA	NA	NA	4
Wayne	NA	NA	NA	15	0	15	NA	NA	NA	15
White	NA	NA	NA	2	0	2	NA	NA	NA	2
Williamson	5	0	5	2	0	2	NA	NA	NA	7
Winnebago	1	0	1	NA	NA	NA	NA	NA	NA	1
Species Total	261	0	261	282	4	286	104	0	104	651

NA = counties were not sampled using surveillance strategy.

**Table 4.** Total tick numbers by county for *I. scapularis* adults and nymphs from each surveillance strategy

	Passive surveillance			Special active			Systematic active			
County	Adult	Nymph	Total	Adult	Nymph	Total	Adult	Nymph	Total	Species total
Alexander	NA	NA	NA	0	0	0	NA	NA	NA	0
Bond	NA	NA	NA	0	0	0	NA	NA	NA	0
Calhoun	NA	NA	NA	0	0	0	NA	NA	NA	0
Champaign	0	0	0	NA	NA	NA	1	3	4	4
Clark	0	0	0	0	0	0	NA	NA	NA	0
Clay	NA	NA	NA	0	0	0	NA	NA	NA	0
Clinton	NA	NA	NA	0	0	0	NA	NA	NA	0
Cook	1	2	3	NA	NA	NA	NA	NA	NA	3
Crawford	NA	NA	NA	0	1	1	NA	NA	NA	1
Cumberland	NA	NA	NA	0	1	1	NA	NA	NA	1
DuPage	3	0	3	NA	NA	NA	NA	NA	NA	3
Edwards	0	0	0	0	1	1	NA	NA	NA	1
Effingham	NA	NA	NA	0	0	0	NA	NA	NA	0
Fayette	NA	NA	NA	0	0	0	NA	NA	NA	0
Franklin	NA	NA	NA	0	0	0	NA	NA	NA	0
Gallatin	NA	NA	NA	0	0	0	NA	NA	NA	0
Hamilton	NA	NA	NA	0	0	0	NA	NA	NA	0
Hardin	NA	NA	NA	0	0	0	NA	NA	NA	0
Henderson	1	0	1	NA	NA	NA	NA	NA	NA	1
Iroquois	NA	NA	NA	1	1	1	NA	NA	NA	1
Jackson	6	0	6	0	0	0	NA	NA	NA	6
Jasper	0	0	0	0	0	0	NA	NA	NA	0
Jefferson	NA	NA	NA	0	0	0	NA	NA	NA	0
Jersey	NA	NA	NA	0	0	0	NA	NA	NA	0
Johnson	0	0	0	0	0	0	NA	NA	NA	0
Kankakee	0	0	0	NA	NA	NA	NA	NA	NA	0
Lake	0	0	0	NA	NA	NA	NA	NA	NA	0
Lawrence	0	0	0	0	1	1	NA	NA	NA	1
Macon	NA	NA	NA	NA	NA	NA	4	3	7	7
Macoupin	0	0	0	0	1	1	NA	NA	NA	1
Madison	NA	NA	NA	0	0	0	NA	NA	NA	0
Marion	0	0	0	0	0	0	NA	NA	NA	0
Massac	0	0	0	0	0	0	NA	NA	NA	0
McDonough	0	0	0	NA	NA	NA	NA	NA	NA	0
McHenry	4	2	6	NA	NA	NA	NA	NA	NA	6
Monroe	NA	NA	NA	0	0	0	NA	NA	NA	0
Montgomery	NA	NA	NA	0	2	2	NA	NA	NA	2
Perry	0	0	0	0	2	2	NA	NA	NA	2
Piatt	NA	NA	NA	NA	NA	NA	7	14	21	21
Pike	NA	NA	NA	0	0	0	NA	NA	NA	0
Pope	0	0	0	0	0	0	NA	NA	NA	0
Pulaski	NA	NA	NA	0	0	0	NA	NA	NA	0
Randolph	0	0	0	0	0	0	NA	NA	NA	0
Saline	NA	NA	NA	0	0	0	NA	NA	NA	0
Shelby	0	0	0	0	0	0	NA	NA	NA	0
St. Clair	0	0	0	0	0	0	NA	NA	NA	0
Tazewell	NA	NA	NA	0	0	0	NA	NA	NA	0
Union	0	0	0	0	0	0	NA	NA	NA	0
Vermilion	0	1	1	NA	NA	NA	NA	NA	NA	1
Wabash	0	0	0	0	0	0	NA	NA	NA	0
Washington	NA	NA	NA	0	0	0	NA	NA	NA	0
Wayne	NA	NA	NA	0	0	0	NA	NA	NA	0
White	NA	NA	NA	0	0	0	NA	NA	NA	0
Williamson	0	0	0	0	0	0	NA	NA	NA	0
Winnebago	1	0	1	NA	NA	NA	NA	NA	NA	1
Species Total	16	5	21	0	10	10	12	20	32	63

NA = counties were not sampled using surveillance strategy.

**Fig. 5.**
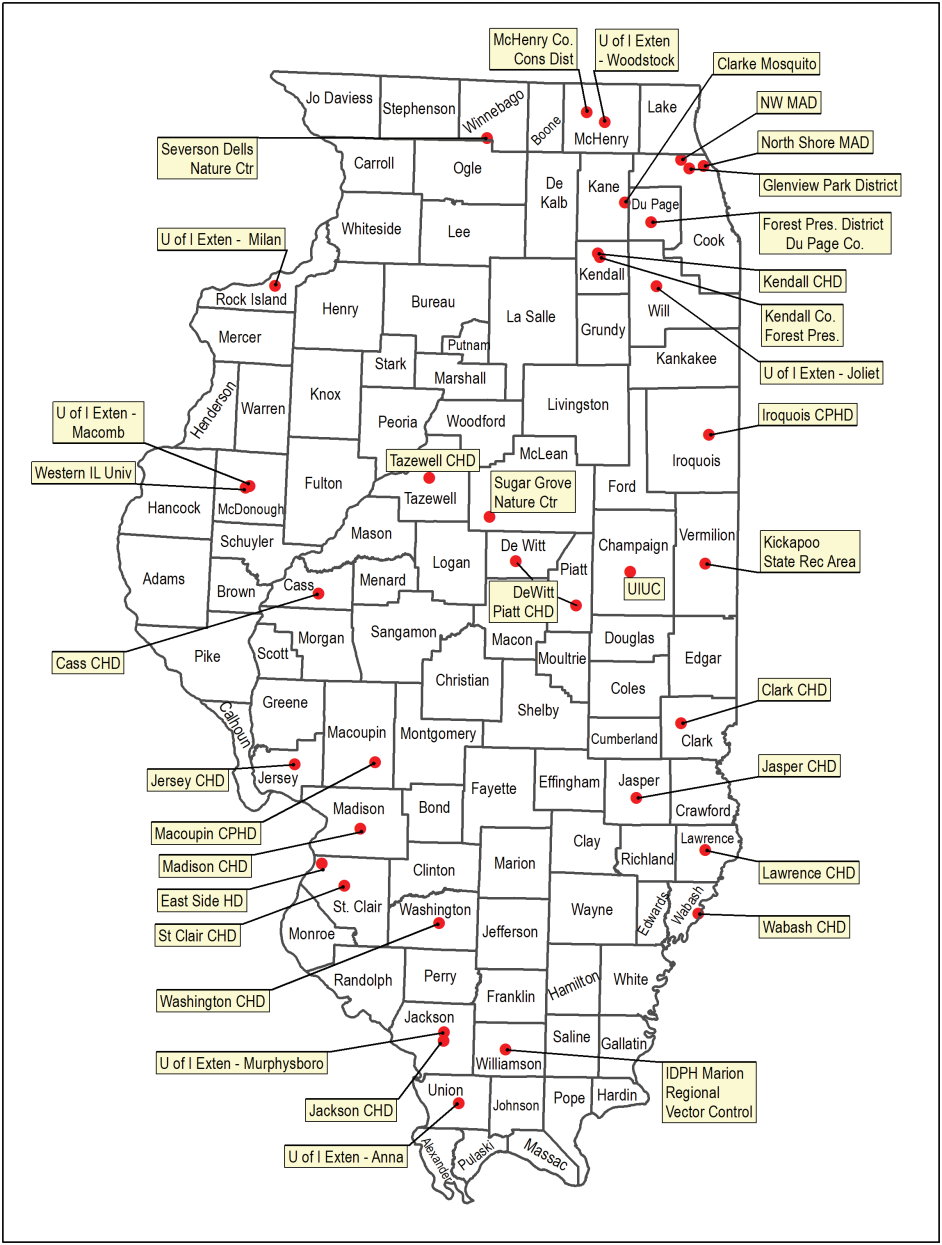
Locations of 2018 passive collection hubs.

During the 2018 passive surveillance program, a total of 1,000 kits were distributed to 35 hubs, covering 42 counties (Fig. 5). There were 131 completed kits (13.1%) from 20 hubs (57.1%) covering 35 counties returned to UIUC. Forty-nine of the completed kits were from University of Illinois Extension offices, 22 each from hubs associated with natural areas or local health departments, 20 from universities, 12 from hubs associated with vector abatement, and 6 from IDPH. Repellent was used 42% of the collection days by participants. Of the 131 kits returned, 29 were returned with zero ticks. In the other 102, the average number of ticks found per kit was six. Passive surveillance by itself accounted for: seven counties switching from reported to established with *D. variabilis*; six counties from reported to established with *A. americanum*; one county from reported to established with *I. scapularis*; one county from no report to established with *A. americanum*; and one county (Champaign) from no report to reported with *A. maculatum*.

Systematic surveillance accounted for ~8% of the total adult and nymphal ticks collected in 2018. Most of these ticks were collected in Macon (101), followed by Piatt (36) and Champaign (5) counties. *Ixodes scapularis* were collected at all six natural areas (*n* = 33: 12 adults, 20 nymphs, and 1 larva). *Dermacentor variabilis* were collected in five natural areas (*n* = 104 adults), and *A. americanum* (*n* = 3; one of each life stage) as well as *A. maculatum* (*n* = 4 adults) were collected at two natural areas. The status of all four tick species changed in Macon county.

Most adult and nymphal ticks collected in 2018 were from special collections (69%). *Amblyomma americanum* was the most abundant (*n* = 972: 204 adults and 768 nymphs) followed by *D. variabilis* (*n* = 286: 282 adults and 4 nymphs). Ten nymphal *I. scapularis* and two adult *A. maculatum* were also collected. Special collections led to seven counties switching from reported to established with *D. variabilis*, while one went from no report to established and one went from no report to reported. *Amblyomma americanum* status changes included: 14 counties from reported to established, 4 counties from no report to established, and 3 counties from no report to reported. *Ixodes scapularis* status changed in one county that went from no report to reported. *Amblyomma maculatum* status changed in two counties (Cumberland and Effingham) from no report to reported.

**Fig. 6.**
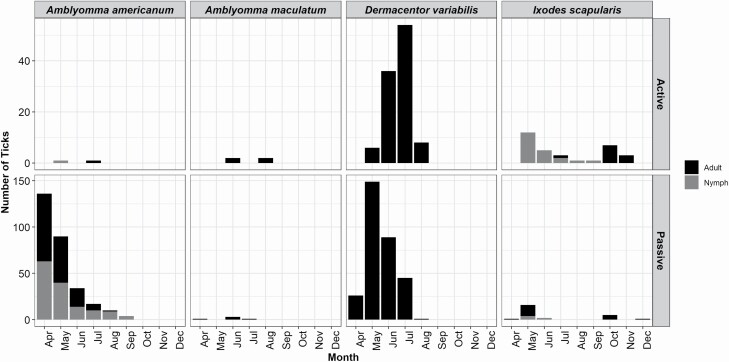
Phenology of passive and active systematic collections in 2018.

Collection dates for the passive surveillance ranged from April 11th to December 18th with ~97% of collections between May and July (Fig. 6). Collection dates for the active systematic surveillance ranged from May 22nd to November 8th with ~92% of collections between May and August (Fig. 6). One nymphal and one adult *A. americanum* were found during systematic surveillance in May and July, respectively. About 95% of adult and ~84% of nymph *A. americanum* collected by passive surveillance were collected between April and June. The majority of *A. maculatum* collected by both passive and systematic surveillance were found in June. *Dermacentor variabilis* adults were collected most frequently in May–June for passive and June to July for systematic surveillance. Passive surveillance found the most (~63%) adult *I. scapularis* during the month of May, while systematic found the most (~64%) in October. All the *I. scapularis* nymphs collected through passive surveillance and the majority of those collected through systematic (~81%) were found in May and June.

## Discussion

While Illinois is recognized as being on the leading edge of range expansion for multiple vector tick species and emerging tick-borne pathogens, prior to 2018, it lacked a statewide surveillance network (Rydzewski et al. 2011, Springer et al. 2014, Lockwood et al. 2018, Phillips et al. 2020, Tuten et al. 2020). The goals of the I-TICK program were to increase and coordinate surveillance and obtain additional information on tick species distributions within the state. This is the first program within the state to use both passive and active tick surveillance strategies to maximize the number of counties with tick collection efforts and provide the information needed to determine tick distribution status.

The 2018 year of the passive surveillance program was considered a building year for the program. During the recruitment process, multiple local health departments recognized the need for more tick surveillance within Illinois but stated shortages in personnel and resources made it difficult for them to have active surveillance programs (Mader et al. 2020). For these reasons, local health departments were very supportive of the passive surveillance program and made up many of the hubs. Recruitment through the University of Illinois Extension offices was also key in the first year of the program. Not only did the offices work as hub locations that covered multiple counties, but they also provided many active participants. One-third of the returned kits came from U of I Extension; active Master Naturalist and Master Gardner programs through these offices could account for increased participation compared to other hubs.

The goals of the 2018 year were to establish a hub in 30 counties throughout the state, receive at least one completed kit from each hub, and have 250 completed kits returned. While we were able to establish over 30 hubs, not every hub returned a completed kit and only 131 kits were returned overall. Despite the low numbers of completed kits, ~30% of the total adult and nymphal ticks collected in 2018 came from the passive surveillance program. Additionally, the program was able to provide surveillance data from 10 counties which were not reached by active surveillance due to lack of time and resources. As with similar studies using citizen science/passive surveillance, there are some limitations with our collection method: awareness and distribution of hubs was not balanced throughout the state, a complete travel history was not collected, and demographic information about participants was lacking (Koffi et al. 2012, Eisen and Paddock 2020, Eisen and Eisen 2021). To help control for the lack of travel history, we did take the additional step of removing engorged ticks from determining changes to establishment status. Moreover, while there have been many successful passive surveillance programs, most of them had a single main organization receiving samples straight from the public and/or from healthcare-related partners (i.e., medical offices, veterinary clinics, laboratory records) (Rand et al. 2007, Xu et al. 2016, Tulloch et al. 2017, Nieto et al. 2018, Ripoche et al. 2018, Kopsco et al. 2020). Our program worked to build collaborations between academic, governmental, and public agencies and provided the public with an opportunity to use and work with those agencies. It also developed resources to answer the public's questions about and increase their awareness of the ticks in their area. The no-cost tick collection kits allowed for preservation of samples until being shipped back to the university and removed monetary boundaries of participants.

We used both timed (special collections) and distance (systematic collections) dragging techniques in our active surveillance strategies. The special collections maximized the number of counties surveyed in the study period. The special collections helped to collect as many ticks as possible in the locations surveyed within the shortest amount of time possible. Results from the special collections can be used in future studies to decide on setting up long-term tick surveillance sites. The systematic collections maximized the opportunity for semi-permanent sites where collections could take place both on and off trails and can be used for future tick phenology studies.

Comparing the three strategies, we were able to find more ticks in more counties using the passive and special collections. This is most likely due to the location of the sampling sites. These two methods were able to cover a broader area especially in the southern parts of Illinois, where the habitat is more desirable for ticks and their preferred hosts (Guerra et al. 2002, James et al. 2015, Springer et al. 2015). For example, much of the area of the five counties with the largest number of ticks from special collections is covered by the Shawnee National Forest. In contrast to this, most of the landcover in the counties chosen for the systematic collections is cropland. Probably due to these same reasons, most of the county status changes were from the special collections followed by the passive collections. The passive collections had the additional benefit of providing information on tick distributions in the northern parts of the state which we were not able to reach with the active methods. Twelve counties that were surveyed using passive and one of the active surveillance methods did not agree on tick status changes between methods. These differences can be attributed to the timing and/or broader coverage area of the passive surveillance collections as well as differences in collection methods.

Tick seasonality varies based on species and life stage. The passive collections in the southern portions of the state occurred between April and December while the only active surveillance was performed in June. Tick seasonality matched between the passive and active systematic surveillance strategies and published surveillance literature (Siegel et al. 1991, Burg 2001, Teel et al. 2010, Gilliam et al. 2018). With passive surveillance, ticks are collected by the participants after they have walked through a natural area, while the active surveillance used dragging techniques to collect ticks. Walking samples can be an effective way of collecting adult life stages, but is not as effective for the immature stages (Ginsberg and Ewing 1989). Additionally, passive participates may have a harder time finding and recognizing nymphal as compared to adult ticks.

Historic tick data have shown the distributions of the dominant tick vectors within Illinois vary by species (Gilliam et al. 2020). *Dermacentor variabilis* is the most widespread species within the state, but its known distribution is incomplete. One reason for this could be the lack of surveillance focus6ing on it (Lehane et al. 2020). Our research supports this theory of underreporting. Our active surveillance included collection in grassland habitats and along trails, the preferred environment for *D. variabilis* (McNemee et al. 2003, Eisen et al. 2017b). By combining the three different surveillance strategies discussed in this paper and not focusing on collecting one specific species, we were able to reveal a 1.56-fold increase in county establishment of *D. variabilis*.

A total of 29 counties changed status for *A. americanum* through the 2018 surveillance efforts. Most of the 2018 status changes for this species were in the southern region of the state, supporting its northward range expansions over the past 20 yr (Eisen et al. 2017b, Gilliam et al. 2020). The aggressive nature of this species toward humans and domesticated animals as well as increasing abundance of its primary host, the white-tailed deer, contribute to its rapid expansion in this area (Childs and Paddock 2003, Springer et al. 2014). The lack of prior surveillance targeting this species is also likely a contributing factor to the numerous changes we documented.

The smallest number of status changes was with *I. scapularis*, for which the status of three counties was changed by the I-TICK program in 2018. We have more information, compared to the other tick species, about the geographical distribution of this species within Illinois thanks to multiple research efforts in the northern half of the state (Rydzewski et al. 2011, Hamer et al. 2012, Schneider et al. 2015, Eisen et al. 2016). The first year of I-TICK focused more on the southern part of the state which may account for all three status changes occurring in this region. The low numbers of *I. scapularis* collected in 2018 may be related to the method of the active surveillance. The special collections in Southern Illinois were in June. While this is associated with peak nymphal questing times for *I. scapularis*; multiple studies have found it difficult to collect nymphs by drag sampling south of the 39th parallel, which includes the targeted area (Diuk-Wasser et al. 2006, Goddard and Piesman 2006). Additionally, we did not drag during the same time of day in all locations during this special collection due to time constraints and inclement weather. *Ixodes scapularis* prefer to quest during the early morning hours, while *A. americanum* prefer the afternoon (Schulze and Jordan 2003). Differences in the host-seeking behavior of *I. scapularis* and *A. americanum* could also play a role in the low numbers. *A. americanum* will use both hunting and ambushing techniques to find potential hosts, so that encounters with this species occur more frequently than for *I. scapularis*, which relies solely on ambushing (Schulze et al. 2005). It may also be that *I. scapularis* have not yet expanded across the state.

Unexpectedly, we collected *A. maculatum* in six counties. While *A. maculatum* has been reported before in Illinois, the combination of all three collection strategies resulted in finding it for the first time in four counties (Bishopp and Trembley 1945, Gilliam et al. 2020, Phillips et al. 2020). *Amblyomma maculatum* may be established farther north within Illinois than previously realized. Furthermore, increased abundance of *A. maculatum* could be one explanatory factor for the increased incidence of Spotted Fever Group Rickettsioses (SFGR) within Illinois, as *A. maculatum* is a known vector of rickettsial pathogens (Phillips et al. 2020). Research focusing on counties with underreporting of ticks and analysis of risk factors for SFGR in Illinois are needed to confirm this hypothesis. Recognizing the need for more information on tick distributions and disease agents in Illinois, a coordinated active statewide and state-funded tick surveillance program, based on using human case data and environmental targeting, began in July 2019 (Tuten and Stone, in preparation).

The first year of the I-TICK program worked to build a network of collaborations and partnerships to support future tick surveillance efforts within Illinois. It highlighted three methods for tick surveillance and the benefits of using them in combination to determine geographic spread of ticks, pinpoint locations in need of more surveillance, and help with long-term efforts that support phenology studies. The information it provided can be used by public health agencies to update tick distribution maps and better inform the public on tick exposure risk within Illinois. Future directions for the program will focus on expanding the passive surveillance network into areas of Illinois that were underrepresented during the first year and focusing systematic active surveillance efforts in new areas based on the results from the first year.

## Supplementary Material

tjab031_suppl_Supplementary_Table
