# Supplementary material for: Comparing Contributions of Passive and Active Tick Collection Methods to Determine Establishment of Ticks of Public Health Concern Within Illinois
Source: J Med Entomol. 2021 Apr 15;58(4):1849–64. doi: 10.1093/jme/tjab031 (PMC8285025; doi:10.1093/jme/tjab031)
Supplement: tjab031_suppl_Supplementary_Table [file tjab031_suppl_supplementary_table.docx]

Supplementary Table:

Supplementary Table 1: Passive collection effort per county

| **County** | **# Collections Days** | **Hours in Field** | **Number of Ticks *** | **Effort**  **(field hour/tick)** |
| --- | --- | --- | --- | --- |
| **Champaign** | 85 | 199.67 | 16 | 12.48 |
| **Clark** | 6 | 8.00 | 7 | 1.14 |
| **Cook** | 66 | 391.25 | 63 | 6.21 |
| **DuPage** | 26 | 121.25 | 7 | 17.32 |
| **Edwards** | 9 | 23.00 | 11 | 2.09 |
| **Fulton** | 1 | 3.00 | 0 | NA |
| **Hardin** | 1 | 9.00 | 0 | NA |
| **Henderson** | 4 | 24.00 | 6 | 4.00 |
| **Iroquois** | 2 | 1.00 | 0 | NA |
| **Jackson** | 107 | 274.83 | 89 | 3.09 |
| **Jasper** | 11 | 5.58 | 12 | 0.47 |
| **Johnson** | 16 | 42.50 | 7 | 6.07 |
| **Kankakee** | 5 | 17.00 | 2 | 8.50 |
| **Lake** | 12 | 81.00 | 4 | 20.25 |
| **Lawrence** | 13 | 16.25 | 31 | 0.52 |
| **Macoupin** | 12 | 107.50 | 14 | 7.68 |
| **Marion** | 3 | 13.00 | 25 | 0.52 |
| **Massac** | 2 | 4.00 | 2 | 2.00 |
| **McDonough** | 10 | 41.00 | 23 | 1.78 |
| **McHenry** | 34 | 114.13 | 20 | 5.71 |
| **Perry** | 13 | 14.00 | 9 | 1.56 |
| **Pope** | 7 | 29.00 | 5 | 5.80 |
| **Pulaski** | 1 | 2.00 | 0 | NA |
| **Randolph** | 11 | 20.00 | 8 | 2.50 |
| **Richland** | 1 | 1.00 | 0 | NA |
| **Shelby** | 2 | NA | 2 | NA |
| **St. Clair** | 10 | 8.08 | 34 | 0.24 |
| **Union** | 8 | 10.00 | 4 | 2.50 |
| **Vermilion** | 5 | 20.00 | 34 | 0.59 |
| **Wabash** | 5 | 18.00 | 3 | 6.00 |
| **Wayne** | 1 | 12.00 | 0 | NA |
| **Will** | 2 | 16.00 | 0 | NA |
| **Williamson** | 34 | 87.00 | 2 | 43.50 |
| **Grand Total** | 525 | 1734.05 | 436 | 3.98 |

* Adult and nymphal ticks collected via the passive collections for each county
